# Women's Limited Choice and Availability of Modern Contraception at Retail Outlets and Public-Sector Facilities in Luanda, Angola, 2012–2015

**DOI:** 10.9745/GHSP-D-16-00304

**Published:** 2017-02-13

**Authors:** Benjamin Nieto-Andrade, Eva Fidel, Rebecca Simmons, Dana Sievers, Anya Fedorova, Suzanne Bell, Karen Weidert, Ndola Prata

**Affiliations:** a Population Services International/Angola, Luanda, Angola.; b Institute for Reproductive Health at Georgetown University, Washington, DC, USA.; c Population Services International, Washington, DC, USA.; d Johns Hopkins Bloomberg School of Public Health, Baltimore, MD, USA.; e Bixby Center for Population, Health, and Sustainability, University of California, Berkeley, Berkeley, CA, USA.

## Abstract

In Angola, many women want to use family planning but lack access to affordable and preferred methods. This article assesses the link between women's choice and availability of contraceptive methods in Luanda, Angola, drawing on data from 3 surveys: a 2012 survey among women ages 15–49 and 2 retail surveys conducted in 2014 and 2015 among outlets and facilities offering contraceptive methods. Descriptive statistics for women's contraceptive knowledge, use, and preferred methods were stratified by age group. We report the percentage of establishments offering different methods and brands of modern contraception, and the mean price, volume of units sold, and value (Angolan Kwanzas) for each brand. Data from the 2 retail surveys are compared to measure changes in availability over time. Results show that 51% of women reported having an unwanted pregnancy. Less than 40% of women knew about long-acting reversible contraceptives (LARCs). Overall, the method most commonly used was male condoms (32.1%), with a substantial proportion (17.3%) of women not using their preferred contraceptive. Trends in contraceptive use mirror availability: in 2015, condoms were available in 73.6% of outlets/facilities, while LARC methods were available in less than 10%. The availability of different methods also dropped significantly between 2014 and 2015—by up to 15 percentage points—with a subsequent price increase in many brands. To meet women's needs for contraception and make informed choice possible, Angola should reinforce demand creation and contraceptive supply in both the public and private sectors through behavior change programs aimed at both women and providers, improved quality of services, training of health personnel on method options and delivery, and improved supply chain distribution of contraceptives. This will allow women to find the methods and brands that best suit their needs, preferences, and ability to pay.

## Abstract (precis)

Despite high rates of unintended pregnancy, access to a wide range of contraceptive methods, especially injectables and long-acting reversible contraceptives (LARCs), is severely limited in both public and private facilities. Knowledge of contraceptive choices is likewise limited, yet a substantial proportion of women are not using their preferred method among the methods they know of.

## INTRODUCTION

Angola's slow recovery after almost 30 years of civil war has led to the country's unique demographic situation compared with other similarly developed sub-Saharan African nations.^1^^,^^2^ Specifically, women in Angola were less likely to have children during wartime, particularly in areas where the impact was the most intense; the probability of having a child was only 39% in 1994 during the intense war period, compared with 52% in 1996 when fighting was at a stalemate.^3^

The temporary decline in childbearing during the war did not result, however, in a long-term reduction in fertility: in 2014, the total fertility rate was 5.7 births per woman, one of the highest rates in the world.^4^ Geographic differences in fertility are stark, with women in rural areas having an average of 3 more children than women in urban areas.^5^ Over time, the age-specific fertility rate among adolescents increased faster than that of older women. For example, from 2006 to 2011 the age-specific fertility rate among 15–19-year-olds increased from 151 to 188 births per 1,000 women, compared with an increase from 242 to 244 births per 1,000 women among 30–34-year-olds.^6^ High fertility rates are often associated with high maternal and child mortality. In Angola, the infant mortality rate is 96 per 1,000 live births and the maternal mortality ratio is 460 per 100,000 live births.^7^^,^^8^

Not surprisingly, the contraceptive prevalence rate (CPR) is low (17.7% for modern and traditional methods; 12.8% for modern methods only), with significant differences by geography (27% of urban women use contraception vs. 7% of rural women) and income level (28.1% of women in the wealthiest quintile vs. 1.4% of women in the poorest).^5^^,^^8^^,^^9^ Contraceptive use is also associated with education, marital status, and previous childbearing experience. A study in Luanda, the capital of Angola, found that sexually active women who are unmarried, less educated, and have never given birth are less likely to be users of any method of contraception.^10^ More than half of women who were not using contraception at the time of the study reported feeling that contraception was inaccessible, underlying the importance of improving method availability and choice.^10^

Women's preferences for particular methods are based on a multitude of factors, including cost, ease of use, efficacy, side effects (both real and perceived), and partner relationships, among many others factors. Having a full range of family planning options is considered an important component of quality of care and informed choice, resulting in improved uptake of family planning.^11^ Access to more methods increases the odds that a woman will use and continue to use contraception: for each additional method widely available in a country, the percentage of married women using contraception increases by an average of 3.3 percentage points.^12^ At the same time, greater availability of contraceptive brands generates competition in the market, increasing chances of price reduction and improving women's access to contraceptives within the private sector.^13^

Angola's national family planning program began in the mid-1980s with the goal of improving maternal health by spacing pregnancies.^15^ The program promised to offer free family planning services through state-run public clinics (maternity hospitals, health centers, and health posts), but implementation has been difficult due to the limited number of trained providers and inconsistent supply of contraceptive commodities.^2^^,^^15^^–^^17^ When the public sector experiences stock-outs of short-acting methods, women might be directed to get contraceptives from the private sector, which is comprised of small local pharmacies (*boteco*), modern pharmacies (e.g., Mecofarma), and private clinics.^18^^,^^19^ In the case of condoms, the private sector also includes non-traditional establishments such as local stores, markets, gas stations, and hotels.

The public sector obtains contraceptives through donations from the United States Agency for International Development (USAID) or the United Nations Population Fund (UNFPA). Specific brands of oral contraceptive pills (Microgynon, Microlut), injectables (Depo-provera), and implants (Jadelle) are donated exclusively for the public sector for free. In 2015, the private sector generally obtained contraceptives from wholesalers (including Princefarma, Shalina, Farwell, and Ecofarma, among others) who import products mostly from Europe and Asia and, in some cases, from Angolan border countries.^20^^,^^21^ The products in the private sector include a wide range of brands for condoms (Davigra, Kamasutra, Durex, among others), oral contraceptive pills (e.g., Yasmin, Diane35), and oral emergency contraceptives (Pilula S and Norlevo).^20^^,^^21^

Angola's family planning strategy currently centers on strengthening supply so that women can choose from a range of affordable products and services needed to space or limit pregnancies. Angola's National Health Development Plan is bolstering health systems to increase the proportion of facilities offering family planning and better distribute qualified providers.^22^ UNFPA helped obtain more than 60% of the country's family planning commodities, and its program for 2015–2019 allocates US$12 million to strengthen the supply chain and integrate activities for family planning, maternal health, and prevention of sexually transmitted infections (STIs) including HIV.^9^ In its 2014–2017 Country Development Cooperation Strategy, USAID will use a market-based approach to support the local commercial sector to supply a suite of contraceptive products to women at different income levels.^22^

Based on the existing literature, little is known about the availability of different contraceptive methods in Angola or the choices women make. This article helps to contribute to the discussion by presenting results from a population-based survey (2012) and two retail studies (2014 and 2015), all conducted in Luanda, Angola, to understand women's contraceptive choices and their link with contraceptive availability in the market.

Little is known about the availability of a range of contraceptive methods in Angola or the choices women make.

## MATERIALS AND METHODS

### Data Sources

This analysis draws on data from 3 surveys conducted by Population Services International (PSI) to understand women's choices and availability of contraception.

**Family Planning Survey 2012.** The 2012 study, conducted in collaboration with the Bixby Center at the University of California Berkeley, surveyed women ages 15–49 in Luanda province between October and November 2012. The purpose of the survey was to assess contraceptive use and fertility preferences, as well as barriers to and drivers of contraceptive use. The questionnaire was based on a standard PSI questionnaire that explores the opportunity, ability, and motivational variables to use contraception, and it borrowed elements of the family planning section of the Angola Malaria Indicator Survey 2011 and of the Women's Questionnaire supplement to the Demographic and Health Surveys (DHS) 2008–2013.^6^^,^^23^ A total sample of 1,545 women completed the survey through multistage random sampling. First, the sample size was distributed proportionally to the size of each municipality. Then a number of sampling points (churches, hospitals, gas stations, etc.) were randomly selected in each municipality from a list created for that purpose. The number of sampling points chosen per municipality varied according to its population size. Within each of these sampling points, a fixed number of households was selected. One woman within the age criteria was then randomly chosen and invited to participate. PSI hired a research marketing agency, SINFIC (*Sistemas de Informação Industriais e Consultoria*), to conduct the fieldwork.

A 2012 population-based study in Luanda province assessed contraceptive use and fertility preferences as well as barriers to and drivers of contraceptive use.

**Retail Survey 2014.** PSI conducted an audit survey among retail stores and health facilities in Luanda province from June to September 2014 to learn about the availability of different contraceptive methods, the brands available for each method, the units sold, the price, and stock-out problems. SINFIC was also hired to conduct the fieldwork for this study. The survey included a census of all establishments selling or distributing at least 1 type of contraceptive in the entire province of Luanda (301 *bairros*), including pharmacies, drug dispensing units in public hospitals, supermarkets, grocery stores, kiosks, gas stations, bars/discos, and hotels. Of the 2,173 establishments identified, 1,833 completed the interview (84.3% response rate). Reasons for not participating were (1) establishment was closed during fieldwork, or (2) absence of manager or owner who could provide permission. Three visits were made to the establishment before desisting from the interview. Establishments participating in the study were similar to those not participating in terms of establishment type. Of the final sample, 2.8% of the establishments were from the public sector (96.1% drug dispensing units at public health centers and 3.9% NGOs), while 97.2% were private-sector outlets (76.6% *boteco*, 10.0% modern pharmacies, 6.7% pharmacies of private clinics, and 6.0% other type of outlet such as kiosks, local stores, street vendors, hotels, and gas stations).

**Retail Survey 2015.** This second round of the retail study also measured contraceptive availability, brand market share, and price. It used the same data collection instrument as in 2014, but unlike the first round, it selected only 50 neighborhoods (*bairros*) at random out of a total of 301 in the entire province. Within those selected neighborhoods, a census of all establishments was conducted. Based on the levels of contraceptive availability detected in 2014, sample sizes for outlets were calculated for each method provided, assuming a 95% confidence interval, 84.0% response rate, 80% power, 2-sided significance tests, and a design effect of 2. The number of completed interviews needed for each method (after discounting the 16.0% non-response rate), was (1) 344 establishments for condoms, (2) 501 establishments for injectable methods, (3) 769 establishments for oral contraceptive pills, and (4) 820 for emergency oral contraceptive pills. Details of the sampling formula can be found elsewhere.^24^^,^^25^ Fieldwork was conducted by PSI/Angola. A total of 957 establishments were identified and invited to participate; 766 completed the interview (80.0% response rate). No notable differences were found in the type of establishments that participated in the study compared with those that did not. Of the 766 establishments included in this analysis, 3.5% were public-sector outlets (85.2% drug dispensing units at public health centers, 7.4% public maternities, and 7.4% NGOs), while 96.5% were private-sector outlets (62.0% *boteco*, 19.5% modern pharmacies, 3.5% pharmacies of private clinics, and 15.0% other type of outlet).

Two audit surveys among retail stores and health facilities conducted in 2014 and 2015 measured contraceptive availability, brand market share, and price.

### Data Analysis

**Family Planning Survey 2012.** Descriptive statistics are presented in this article, including: *sociodemographics* of the sample, *pregnancy intention* (measured by whether women wanted their last pregnancy at the time it happened, wanted it later, or did not want more children at all), *contraceptive knowledge* (prompted), *contraceptive use* (the percentage of sexually active women using modern methods), as well as the *unmet need for preferred contraception* (defined as the percentage of sexually active, fecund women who are currently using contraception but report that their current method is not their preferred method). Main results are presented for the overall sample and by age group. Given that adolescent women generally face greater barriers in accessing reproductive health services, 2 age groups were created from the sample of young women: ages 15–19 and ages 20–24. Women aged 25 and older comprised the final age group.

**Retail Surveys 2014 and 2015.** Availability of contraceptive methods on the market was calculated for each year, defined as the percentage of establishments that reported selling or distributing different types of modern contraceptive methods. Independent sample *t* tests were produced to measure changes over time for the overall samples and for the private and public sector, separately.

For 2015, market share indicators were produced, defined as the proportion that each brand represents within its respective contraceptive category in terms of volume (number of units sold) and value (Angolan Kwanzas: AKZ), measured during the 30 days prior to the survey. No data were presented for 2014 given that the dominant contraceptive brands remained so in 2015. Availability of brands was also calculated based on proportion of outlets having specific brands within the outlets selling or distributing the corresponding contraceptive category.

Finally, mean price per brand was reported for each year with its 95% confidence intervals, in order to measure significant changes over time. This was calculated as the arithmetic average of price provided by establishments. This indicator is presented in local currency, rather than US dollars, to better reflect changes in prices and its impact on Angolan women: recent depreciation of AKZ with respect to US dollars could hide the real cost of contraceptives to consumers if US currency is used. The exchange rate as of October 30, 2014, was US$1 for 99AKZ.^26^

### Ethical Considerations

The family planning study conducted in 2012 received ethical approval from the Berkeley Center for Protection of Human Subjects (CPHS # 2011-08-3521) at the University of California Berkeley, and from the Ethical Committee at the Public Health Institute in Luanda, Angola. The retail study in 2014 was commissioned to an external agency (SINFIC) and followed its internal ethics processes, which consisted of providing ethics training to its interviewers to minimize any potential risk in breach of confidentiality and to assure justice, beneficence, and respect during the data collection process; interviewers also signed a pledge of confidentiality. The retail study in 2015, conducted by PSI/Angola, went through PSI's ethics committee, which deemed the study to be not human subjects' research as it was a survey about product availability. Interviewers still received training in human subjects to ensure compliance with ethics principles.

## RESULTS

### Women's Fertility Preferences and Contraceptive Use

Results from the 2012 Family Planning Survey show a relatively young female population (median age of 24 years), with more than half having completed high school (Table 1). Most of the women aged 20–24 reported being single (81.7%) and were sexually active (90.9%). Although half of them had already been pregnant, the majority (72.2%) either did not plan or did not want their last pregnancy. Younger women wanted fewer children: women aged 20–24 wanted an average of 3.7 children, while women 25 years and older desired 4.3 children.

**TABLE 1.** Demographic and Behavioral Characteristics of Women of Reproductive Age in Luanda, Angola, 2012

Variables	Age Groups			
	15–19 (N=451)	20–24 (N=361)	≥25 (N=729)	All (N=1545)
***Sociodemographics***				
Age, median	NA	NA	NA	24.0
Marital status				
Single	98.2	81.7	39.4	66.5
Married/cohabiting	1.8	18.3	56.0	31.3
Divorced/widowed	0.0	0.0	4.1	2.2
Education				
High school or less	55.3	28.8	45.3	44.4
More than high school	44.7	71.2	54.7	55.6
***Sexual Behavior and Fertility Preferences***				
Ever had sex	55.0	90.9	91.4	80.6
Ever been pregnant	11.5	49.9	90.4	57.9
Intention to get pregnant at last pregnancy^a^				
Wanted it at that moment	17.3	27.8	57.7	49.3
Wanted it later	53.8	56.1	28.1	35.2
Did not want more children	28.8	16.1	14.3	15.5
Ideal number of children, mean^b^	3.8	3.7	4.3	4.2
Knowledge of modern contraceptives				
Condoms	96.7	95.6	93.7	95.0
Oral contraceptive pills	68.9	84.2	87.4	79.7
Injectable	47.2	65.7	84.1	68.9
Female condoms	34.2	42.1	54.7	45.6
IUD	19.1	36.3	54.2	39.6
Implants	18.2	35.7	52.7	38.6
Female sterilization	14.6	26.6	39.4	29.1
Emergency oral contraceptive pills	10.3	21.6	32.8	23.5
Male sterilization	5.3	12.5	22.2	15.0
***Contraceptive Use***^c^				
Current prevalence of any modern contraception	58.5	64.9	55.9	58.7
Current prevalence of:				
Condoms	52.0	46.0	17.7	32.1
Injectables	2.0	6.1	18.8	12.1
Oral contraceptive pills	3.2	11.0	14.1	11.1
Implants	0.4	1.5	2.6	1.9
IUD	0.0	0.0	1.4	0.7
Female sterilization	0.8	0.0	0.8	0.6
Female condoms	0.0	0.3	0.6	0.4
Male sterilization	0.0	0.0	0.0	0.0
Ever used emergency contraceptive pills^d^	1.1	6.4	5.8	4.5
Current contraceptive users not using their preferred method^e^	15.1	20.7	16.0	17.3
Preferred contraceptive method among women not using their preferred method^f^				
Injectables	13.6	25.0	31.0	25.6
Condom	31.8	22.7	8.6	17.6
Implants	13.6	4.6	27.6	16.8
Oral contraceptive pills	4.6	13.6	12.1	12.0
Rhythm method	4.6	0.0	1.7	1.6
Female condom	4.6	0.0	0.0	0.8
Female sterilization	0.0	0.0	1.7	0.8
None	13.6	20.5	0.0	9.6
Other	13.6	13.6	17.2	15.2

Abbreviation: IUD, intrauterine device.

All data are reported as percentages unless otherwise noted.

a Among women ever pregnant (N=52 among 15–19-year-olds; N=180 among 20–24-year-olds; N=659 among ≥25-year-olds; and N=891 among the entire sample of women).

b Among women who have given birth (N=25 among 15–19-year-olds; N=128 among 20–24-year-olds; N=608 among ≥25-year-olds; and N=764 among the entire sample of women).

c Among women who have had sex (N=248 among 15–19-year-olds; N=328 among 20–24-year-olds; N=666 among ≥25-year-olds; and N=1245 among the entire sample of women).

d The survey did not explicitly include emergency contraceptive pills as an option for current method but included a question on ever use of emergency contraceptive pills.

e Among fecund women who have had sex and who are currently using contraception (N=146 among 15–19-year-olds; N=213 among 20–24-year-olds; N=363 among ≥25-year-olds; and N=722 among the entire sample of women).

f N=22 among 15–19-year-olds; N=44 among 20–24-year-olds; N=58 among ≥25-year-olds; and N=125 among the entire sample of women.

Nearly all women knew about condoms (95.0%) and, to a lesser extent, oral contraceptive pills (79.7%) and injectable contraceptives (68.9%). In contrast, less than half of the respondents knew about long-acting reversible contraceptives (LARCs), which consist of intrauterine devices (IUDs) (39.6%) and implants (38.6%). Knowledge of LARCs was extremely low in the youngest age group (15–19): only 19.1% knew about IUDs and 18.2% knew about implants.

The majority of women knew about condoms, pills, and injectables, but less than half knew about IUDs or implants.

While the overall contraceptive prevalence rate was relatively high among the study participants (58.7%), most of it was due to the reported reliance on condoms, with little to no use of other methods, especially among the younger age groups. For example, 52.0% of women 15–19 years old reported using condoms as a family planning method, while only 3.2% reported using oral contraceptive pills and 2.0% injectable contraceptives. Almost no young respondents used implants (0.4%) or IUDs (0.0%). A similar pattern was observed in the 20–24 year age group.

Although contraceptive prevalence was relatively high (59%) among survey respondents, most of it was due to the reported reliance on condoms.

There was some discrepancy between actual and preferred contraceptive method use: women were looking for a wider range of alternatives than what they actually had. Overall, 17.3% of women currently using contraception reported not using their preferred method. Of these women, the most commonly reported preferred method was injectable contraceptives (25.6%), followed by condoms (17.6%) and implants (16.8%).

17% of women currently using contraceptive reported not using their preferred women.

In summary, results from the 2012 Family Planning Survey among women in Luanda illustrate a high percentage of unwanted pregnancy and a lack of knowledge about the full array of contraceptive methods, particularly LARCs, which is especially true among younger cohorts. There is a heavy reliance on condoms as a family planning method, and there is a substantial percentage of women who are not currently using their preferred contraceptive method.

### Contraceptive Availability in Luanda

Data from the 2014 and 2015 Retail Surveys showed limited choice for contraceptive methods in Luanda, with a decline in availability over that period of time (Table 2). Although 85.9% of all outlets reported having at least 1 method of contraception in 2015, when the information was analyzed by method type, it was clear that women did not have many contraceptive options. In both years, the most widely available contraceptive method on the market was the male condom, present in 81.4% of the outlets visited in 2014 and in 73.6% of outlets visited in 2015 (*P*<.001). Following condoms, oral contraceptive pills and emergency contraceptives were the next most available methods; however, during the same period, their availability declined from 58.6% to 43.3% (*P*<.001) and from 42.4% to 34.4% (*P*<.01) of outlets, respectively. Availability of injectable contraceptives declined significantly by 6.0 percentage points to reach a level of 7.3% in 2015 (*P*<.001).

**TABLE 2.** Availability of Contraceptive Methods by Sector, Luanda, Angola, 2014–2015

Method	Private Sector			Public Sector			Total		
	2014 (N=1782)	2015 (N=739)	Difference	2014 (N=51)	2015 (N=27)	Difference	2014 (N=1833)	2015 (N=766)	Difference
Any method^a^	97.6	86.9	−10.7***	84.3	59.3	−25.0*	97.2	85.9	−11.3***
Male condom	82.3	75.1	−7.3***	49.0	33.3	−15.7	81.4	73.6	−7.8***
Oral contraceptive pills^b^	58.7	43.8	−14.9***	54.1	29.6	−24.5*	58.6	43.3	−15.3***
Emergency contraceptive pills^b^	43.3	35.8	−7.5**	11.8	11.1	−0.7	42.4	34.4	−7.5**
Injectables^b^	13.1	6.6	−7.56.5***	19.6	18.5	−1.1	13.3	7.3	−6.0***

All data for 2014 and 2015 reported as percentages; the differences between 2014 and 2015 are percentage points.

*P<.05;

** *P*<.01;

*** *P*<.001.

a Any method includes at least one of the following: condoms, oral contraceptive pills, emergency contraceptive pills, injectable methods, hormonal patches, spermicides, intrauterine devices (IUDs), mini-mola or Essure (a non-surgical permanent method for women), and Vasalgel (a long-acting gel similar to no-scalpel vasectomy but likely more reversible). Less than 5% of the outlets overall reported having hormonal patches, female condoms, spermicides, IUDs, vaginal rings, or implants. Only 0.1% reported providing mini-mola or Essure or Vasalgel.

b Gas stations, hotels, and bars were not included in the calculation of oral contraceptive pills, emergency contraceptive pills, or injectable availability, since those outlets mainly distribute or sell condoms.

Although 86% of all outlets reported having at least 1 contraceptive method in 2015, the most widely available method on the market was the condom.

When comparing the public and the private sectors, we observed a larger decrease in the availability of oral contraceptive pills in the public sector: while it declined 14.9 percentage points in the private sector (from 58.7% in 2014 to 43.8% in 2015; *P*<.001), it dropped 24.5 percentage points in the public sector (from 54.1% to 29.6%; *P*<.05). We did not observe a significant change in the availability of male condoms in the public sector, most likely due to the small number of public-sector facilities. Availability of the male condom in the private sector, however, dropped significantly by 7.3 percentage points. Overall, the male condom was more available in the private sector than in the public sector in both 2014 and 2015 (for example, 75.1% in the private sector versus 33.3% in the public sector in 2015).

A higher percentage of public-sector establishments distributed injectable contraceptives in both years (over 18.0%) compared with the private sector, which dropped from 13.1% in 2014 to 6.6% in 2015 (*P*<.001). All other methods (hormonal patch, female condoms, spermicides, IUDs, vaginal rings, and implants), which are mostly distributed in the public sector, were present in less than 5.0% of all establishments in both years.

### Brand Market Share and Price

The number of brands on the market is also an indicator of the number of choices available to women in terms of quality and price. This section presents a landscape of the market share (volume and value) and price for the most available contraceptive methods in 2015. While condoms, oral contraceptives, and emergency contraceptive pills have multiple brands in the market, each brand individually only represents a small proportion of market. For example, Table 3 shows that only 4 condom brands have a strong individual presence in terms of sales: Sensual, with 22.8% of total volume of the market and 25.6% of the total value (AKZ), followed by Legal (22.0% volume and 16.7% value), Davigra (15.6% volume and 10.6% value), and Durex (11.0% volume and 18.7% value). Together these 4 brands represent over 70.0% of both measures of market share. Each of the more than 40 other condom brands available in the market is present in less than 5% of the outlets.

**TABLE 3.** Brand Market Share by Type of Contraceptive Among Outlets Selling the Respective Contraceptive Method, Luanda, Angola, 2015

Contraceptive Method and Brand	% Outlets Offering the Brand	Units Sold Last Month^a^	% Units	Value (AKZ) Sold Last Month	% Value
**Condoms (N=635 outlets)**					
Sensual	51.8	17,716	22.8	1,295,650	25.6
Legal	37.3	17,092	22.0	845,000	16.7
Davigra	15.6	12,103	15.6	535,530	10.6
Boss Man	11.8	3,153	4.1	176,240	3.5
Kamasutra	9.6	2,193	2.8	6,300	0.1
Control	6.0	4,711	6.1	611,994	12.1
Durex	3.3	8,576	11.0	948,365	18.7
Generic	1.3	7,527	9.7	293,150	5.8
Other brands (+40)	<5.0 each	4,557	5.9	358,795	7.1
Total for condoms	NA	77,628	100.0	5,071,024	100.0
**Oral contraceptive pills (N=357 outlets)**					
Microgynon	76.6	2,885	66.0	1,058,303	40.6
Microlut	27.7	457	10.4	141,394	5.4
Yasmin	8.6	234	5.3	434,366	16.4
Diane 35	7.8	108	2.5	167,442	6.4
Other brands (+10)^b^	<5.0 each	690	15.8	802,642	31.2
Total for oral contraceptive pills	NA	4,374	100.0	2,604,147	100.0
**Emergency contraceptive pills (N=255 outlets)**					
Pilula S	45.4	1,064	38.4	656,100	22.7
Ella	24.3	595	21.5	554,200	19.1
IPL72	20.4	463	16.7	330,700	11.4
Norlevo	14.1	6	0.2	1,086,707	37.5
Other brands (7)^c^	<5.0 each	643	23.2	266,518	9.3
Total for emergency contraceptive pills	NA	2,771	100.0	2,894,225	100.0
**Injectables (N=54 outlets)**					
Depo-provera	83.3	260	97.7	21,700	94.1
Mesignya	1.9	n/a	n/a	n/a	n/a
Other brands	<1.0 each	6	2.3	1,360	5.9
Total for injectables	NA	266	100.0	23,060	100.0

Abbreviation: AKZ, Angolan Kwanzas.

a Units are individual condoms for condoms; cycles for oral contraceptive pills; packs for emergency contraceptive pills; and individual units for injectables.

b Other brands of oral contraceptive pills included Cezarette, Climen, Ella, Gynera, Marvelon, Minygesty, etc.

c Other brands of emergency contraceptive pills included CO-Pill, Plan Fam, Levo 72, etc.

Of the more than 40 condom brands on the market in Luanda, only 4 of the brands have strong presence in terms of sales.

As seen in Table 4, the prices of Sensual and Legal condoms are among the lowest in the market: 73 and 46 AKZ per individual condom in 2015, respectively (about US$0.74 and $0.47, respectively, given an exchange rate of 99 AKZ for US$1).^26^ Between 2014 and 2015, most of these major brands raised their product price to the final consumer, although by comparing the 95% confidence intervals around the mean values in both years, we observed that only in the case of Legal was the difference statistically significant: the confidence intervals each year do not overlap.

**TABLE 4.** Mean Price (Angolan Kwanzas)^a^ for Main Brands of Contraceptives, Luanda, Angola, 2014–2015

Method and Brand [No. of outlets selling the method in 2014, 2015]	Mean Price per Unit^b^ (95% CI)		% Change
	2014	2015	
**Condoms**			
Sensual [N=697; N=359]	69.5 (44.3, 72.7)	73.4 (71.2, 75.6)	+5.6%
Legal [N=529; N=237]	36.1 (33.2, 39.0)	46.8 (42.8, 50.8)	+29.8%^
Davigra [N=267; N=99]	59.5 (55.5, 63.5)	58.9 (45.0, 72.7)	−1.0%
Durex [N=57; N=21]	119.3 (90.8, 147.8)	200.9 (145.1, 256.8)	+68.4%
Boss Man [N=264; N=71]	54.1 (51.2, 57.0)	60.4 (55.7, 65.2)	+11.7%
**Oral contraceptive pills**			
Microgynon [N=840; N=261]	238.8 (231.7, 245.9)	498.5 (448.3, 548.8)	+108.8%^
Microlut [N=365; N=82]	278.8 (264.5, 293.2)	394.8 (346.0, 443.6)	+41.6%^
Yasmin [N=51; N=43]	705.5 (651.8, 759.3)	2300.2 (2017.5, 2583.0)	+226.0%^
Diane 35 [N=69; N=22]	1068.5 (952.9, 1184.0)	1830.7 (1200.3, 2461.1)	+71.3%^
**Emergency contraceptive pills**			
Pilula S [N=325; N=108]	453.4 (427.3, 479.5)	579.2 (536.0, 622.4)	+27.7%^
Ella [N=225; N=63]	699.3 (657.5, 741.1)	959.0 (659.5, 1258.6)	+37.1%^
IPL72 [N=113; N=46]	509.6 (456.0, 563.3)	700.5 (602.8, 798.2)	+37.5%^
Norlevo [N=109; N=30]	2026.9 (1814.3, 2239.4)	2950.9 (2502.3, 3399.6)	+45.6%^
**Injectables**			
Depo-provera [N=213; N=45]	351.0 (321.0, 281.1)	482.2 (323.0, 641.5)	+37.4%^

^ Indicates significant changes based on 95% confidence intervals not overlapping between 2014 and 2015.

a Prices reflect mostly the private sector. According to the National Health Care System, the public sector must offer health services and medicines for free. The exchange rate as of October 30, 2014, was 99 Angolan Kwanzas for US$1.^26^

b Units are individual condoms for condoms; cycles for oral contraceptive pills; packs for emergency contraceptive pills; and individual units for injectables.

Only 2 oral contraceptive pill brands dominate the market: Microgynon was present in 76.6% of establishments selling or distributing oral contraceptive pills, followed by Microlut, which was present in 27.7% of establishments (Table 3). These 2 brands are both government/donor-procured and together represent 76.4% of units (monthly cycles) sold or distributed and 46.0% of the value sold in the market. On average, these 2 brands are the most accessible to the final consumer in terms of price: 499 AKZ and 395 AKZ, respectively, compared with other brands that ranged in price between 1831 AKZ and 2300 AKZ per cycle in 2015 (Table 4). All brands of oral contraceptive pills significantly increased their price to the consumer by more than 30% between 2014 and 2015, as judged by comparing the 95% confidence intervals around the mean value each year.

As with condoms, only 2 of more than 10 brands of oral contraceptive pills dominate the market in Luanda.

Similarly, out of 11 brands of emergency contraceptive pills, 3 non-government-procured brands dominate the market: Pilula S, Ella, and IPL72. These brands together represent 76.6% of units sold and 53.2% of the market value (Table 3). Like oral contraceptive pills, brands of emergency contraceptive pills significantly increased their price from 2014 to 2015 by at least 27% (Table 4).

In summary, data from the 2014 and 2015 Retail Surveys on contraceptives in Luanda show limited availability of contraceptives. With the exception of condoms and oral contraceptive pills, most other types of contraceptive methods are difficult to find. Even oral contraceptive pills and condoms are not found in all establishments, and their overall availability dropped significantly from 2014 to 2015, while significant increases in price to final consumer were observed in many brands. The private sector reported greater availability of condoms, oral contraceptive pills, and emergency contraceptives compared with the public sector, including brands of oral contraceptive pills that are government/donor-procured for the public sector. These brands should only be available in the public sector for free, yet they are present in the private sector offering the lowest prices.

Most types of contraceptive methods are difficult to find in Luanda, with the exception of condoms and pills.

## DISCUSSION

The analysis of the Family Planning Survey 2012 and the Retail Surveys 2014/2015 suggests that limited knowledge and use of a range of contraceptive methods, and the limited availability of LARCs in particular, are interplaying factors shaping the contraceptive market landscape and women's choices in Luanda, Angola. Recognizing that there are other multiple factors affecting the contraceptive landscape in a country, this article focuses only on demand and supply. Our findings suggest that women's lower knowledge of LARC methods (including misconceptions around them) may explain their lower demand, while the limited presence of those methods in the market can also reinforce women's lower knowledge and use.

Limited knowledge and use of a range of methods, along with limited availability of LARCs in particular, are interplaying factors shaping the contraceptive market landscape in Luanda, Angola.

### Limited Knowledge and Use of LARCs

The present analysis shows that there were important differences in knowledge of contraceptive methods among women of reproductive age. Specifically, more women knew about short-acting methods (such as condoms and oral contraceptive pills) than long-acting methods (IUDs and implants). Furthermore, knowledge of LARCs was lower among adolescents than older women, a result corroborated by other studies that have found that awareness of different contraceptive methods varies by age group.^27^^,^^28^ Because limited knowledge of contraceptive methods can be a barrier to contraceptive use, women of reproductive age should have more scientific information about the different contraceptive methods available to them.^28^

Patterns of contraceptive use mirror the levels of knowledge of each method: male condoms was the most used contraceptive method across all age groups, followed at a distance by oral contraceptive pills and injectable contraceptives. Although the use of LARC methods was generally low across all age groups, an even smaller percentage of adolescents used LARCs compared with older women, a pattern found in other studies.^27^^,^^29^ In 2015, a study conducted by Key Research in Luanda showed that short-acting methods were the most widely used methods by women, with condoms as the most-used method (19.1%), followed by injectable methods (12.0%) and oral contraceptives (9.2%). LARC methods were barely used, particularly among young women.^29^ This confirms qualitative findings where young women reported fears of becoming infertile if they used contraceptive methods other than condoms; they all considered that oral contraceptive pills, injections, and especially LARCs were only for women who already had children.^18^ Given the high fertility rate in Angola, the use of LARCs by young women could make a positive impact in reducing not only the fertility rate but also the rates of unintended pregnancy, unsafe abortion, and maternal mortality.

Patterns of contraceptive use in Luanda mirror the levels of knowledge of each method.

### Impact of Limited Availability of Contraceptives on Women's Choices

The present analysis also shows limited availability of contraceptive methods, with the private sector generally performing better than the public sector. Not surprisingly, in the Key Research study a larger proportion of women in Luanda reported obtaining their method from pharmacies, private hospitals, or local stores (64.8%), compared with public hospitals, health posts, and NGOs (34.2%).^29^ The limited availability of contraceptives in the market appears to constrict women's choice of a contraceptive method.^30^ In our study, almost one-fifth of women using contraception reported not using their preferred method. Among the most common reasons for non-use was the method being difficult to obtain or not being available where women receive family planning (data from the 2012 Family Planning Survey, not shown). The Retail Surveys 2014 and 2015 also show that, overall, condoms, oral contraceptives, and emergency contraceptives are more widely available than other types of contraceptives. Even within the public sector, where IUDs or implants are expected to be available, very few outlets reported having them in stock. This is likely a contributing factor explaining why condoms and oral contraceptive pills are the most widely used methods, while LARC methods are among the least known and used in Luanda.

There is limited availability of a range of methods on the market, with the private sector generally performing better than the public sector.

In general, providers do not recommend LARC because of stock-out problems, lack of training, or beliefs that such methods are only for women who already have children.^21^ This reinforces women's own beliefs that condoms are the best methods for childless women because other methods can cause infertility.^18^

Knowledge, use, and availability of the male condom has increased more sharply in sub-Saharan Africa than in other regions of the world, partly because it can be promoted as a dual protection method that can protect against both unwanted pregnancy and HIV.^30^ In Angola, male condoms were promoted for many years more than any other contraceptive method and have been offered for free or sold in a wide range of outlets in Luanda.^31^ Accordingly, the high availability of male condoms stimulates its greater use across all age groups.

Earlier research has shown that greater availability of different contraceptive methods is linked with higher contraceptive prevalence rates.^12^ Ready access to a wide range of methods allows each subgroup of users to find the method that best fulfills their family planning needs.^30^ Thus, it is important for a country to invest in different contraceptive methods in order to increase informed choice and satisfaction among users. Allowing brand competition also helps the market benefit women by increasing the chances of price reduction, and consequently improving women's access to contraceptive methods.

### Role of International Aid and of the Economic Crisis on Contraceptive Market Landscape

According to market data analyzed in this paper, the most available brands of condoms and oral contraceptives are those that have been supported by external donations. In the case of condoms, the brands Sensual and Legal were originally introduced in the Angolan market with support from USAID and DFID (United Kingdom Department for International Development).^32^ After more than 10 years in Angola, these socially marketed, affordably priced brands are successfully competing, as indicated by their high market share in terms of both volume and value. In the case of oral contraceptives, the Microgynon and Microlut brands were introduced with support from USAID and UNFPA to be distributed for free in the public sector.^33^ However, the retail surveys found these brands on sale in the private sector, contrary to their initial purpose. It is not clear how these brands leak into the private sector, but because they offer the lowest price to consumers and compete against commercial brands, there is little space in the market share for other commercial brands to grow: together these 2 brands represent 76.0% of the total volume of oral contraceptives. The leakage also reflects a weak health system with a poor regulatory environment and limited human resources to monitor the supply chain operations and lack of standard operational procedures.^34^

The most available brands of condoms and pills are those that have been supported by donations by external donors.

Market data also show a significant reduction in the proportion of outlets having different types of contraception from 2014 to 2015. The decrease in availability translates into a price increase for many contraceptive brands and represents a step back in women's choices and affordability of different contraceptives, especially in a country where 56.4% of the employed population lives on less than US$2 per day (less than 200 AKZ).^35^ The situation may persist in the current economic context, in which a substantial drop in oil prices (Angola's main income-generating product) has limited government's expenditure on health and the private sector's capacity to import products due to lack of international currency (US dollars).^36^^,^^37^

Reductions in the proportion of outlets having different types of contraception translated into price increases for many brands.

As Angola has shifted from having a low- to middle-income economy, the role of external donor agencies is changing from directly purchasing essential products to serving in a solely technical or advocacy role. As an example, between 2014 and 2015 UNFPA decreased the number of Microgynon and Microlut oral contraceptive pills donated from 349,189 to 285,730 cycles and the number of Jadelle implants from 85,000 to 0.^20^ In this context, the Angolan government will have to increase its direct investment in contraceptive products and play a more active part in satisfying the need for family planning. In 2012, the government budget represented only 10% of the total amount spent on family planning, compared with 30% provided by UNFPA and 60% by USAID.^21^ In its plans for fiscal year 2016, the Ministry of Health forecasted an investment covering 23.0% of the family planning budget, while UNFPA and USAID would provide the remaining 47.0% and 29.0%, respectively.^38^ There is still a long way to go toward self-sufficiency, and Angola should continue to introduce strategies to ensure availability and fair competition of family planning products on the market, while encouraging demand for contraception.

### Market Segmentation: Public and Private Sectors Working Together on Family Planning

In order to achieve universal coverage for family planning, it is important to have cooperation and coordination between the public and private sectors (including civil society, NGOs and donors). While the public sector can provide country leadership by setting regulations, taxes and fees, policies, and quality standards, as well as serving the lowest quintiles through public services, the private sector can reach population sectors that can afford to pay, thereby reducing the burden on the public sector to provide free commodities broadly.

Through market segmentation, the private sector can reach segments of the population with some economic power interested in using family planning, which are not usually the target of the public sector. Preventing leakage of "free" products from the public sector into the private sector (which are later sold at "unfair" prices) will assure that (a) such products actually reach women with limited resources, for whom they are intended, and (b) the private sector invests more to meet the demand of family planning for a segment that can actually pay for it. With an interest in growing its business, the private sector will guarantee the availability of products that represent a margin of profit for them, reducing the chances of stock-out.

Market segmentation would allow the private sector to reach those population segments with some ability to pay for contraception, thereby reducing the burden on the public sector to provide free commodities broadly.

There is the potential to expand and improve the family planning market in the private sector in Angola, yet this potential is not realized and is often overlooked. Thinking in the new Angolan context in which donors are reducing donation of products and are focusing mostly on technical assistance, it is important to consider how best to reinforce the private sector as a complementing component to the overall health system.

### Limitations

There are some limitations in the present analysis that should be highlighted. First, the family planning survey and the retail studies were not conducted during the same year, preventing a direct comparison between contraceptive use and method availability. The 2-year difference raises the question of whether the context was the same when use and availability of contraceptive methods were analyzed. To mitigate this limitation, information on contraceptive use from an OMNIBUS Study conducted by Key Research in Luanda is incorporated in the discussion section. The study was conducted at the same time as the Retail Study 2015. Overall, it showed similar patterns as the Family Planning Study 2012: a relatively high reliance on male condoms and continued low prevalence of LARC methods, especially among young age groups

The small sample size of public-sector facilities in the retail studies also limits the comparative analysis over time of contraceptive availability in public health facilities. Statistical tests are less likely to show significant differences where there actually is a difference. This limitation cannot be overcome; even when conducting a census of public-sector facilities in the province (as in the Retail Study 2014), the number of public facilities is still limited.

Finally, the required sample size for emergency contraceptive pills was not sufficiently large enough to measure a change of at least 10 percentage points over time, so analysis about statistically significant changes in the availability of this method should be made with caution. The data serve though to understand the tendency over time in the availability of this method, which follows the same tendency reported in this article for other methods.

## CONCLUSIONS

National data show high levels of fertility in Angola: almost 6 children per women at the end of her reproductive life, with the adolescent fertility rate increasing over time.^4^^,^^6^ Many Angolan women want to space or limit childbirth, but they have few real opportunities to do so. This paper shows that in Luanda province, the capital of Angola, half of women did not want their last pregnancy. On average, the ideal number of children reported in this paper (4.2) is lower than the actual number of children born to women at the end of their reproductive life in Luanda (5.5).^39^

Male condoms and oral contraceptive pills are the most available methods in Luanda and the most used methods across all age groups. In contrast, few women rely on implants or IUDs, at least in part because LARC methods are less available. Similar to other research in family planning, we can infer that the availability or unavailability of a contraceptive method influences its use (or non-use). Besides availability, this article finds that other factors such as age and awareness of a contraceptive method were also linked to contraceptive choice and use.

In order to meet the latent demand for contraception, it is necessary not only to ensure the availability and affordability of contraceptives currently on the market, but also to expand the range of options for women and to guarantee women's real access to sexual and reproductive health products and services. To achieve that goal, it is important to take actions toward:

- Improving women's knowledge about all contraceptive methods, including their characteristics and potential side effects, as well as reducing current myths and misconceptions about use of contraception, allowing women to make informed choices
- Increasing the availability of different contraceptive methods in the private and public sector with an adequate segregation of the market; for instance, making generic or less expensive brands available in public sector, while selling more expensive brands in private pharmacies or drug shops
- Improving the efficiency of supply chain distribution to prevent stock-outs in the market, while simultaneously strengthening the regulatory health system, to avoid leakage from the public to the private sector, and helping women from low socioeconomic sectors to have real access to free contraception within the public sector
- Assuring a self-sustained market, where supply meets—and at the same time enhances—demand, with little dependence on donations from international organizations
- Improving family planning services by eliminating provider's own misconceptions (only women who already had children should use contraception due to risks of infertility); also through counseling and provision of all type of methods, including LARC methods
- Advocating the inclusion of all LARC methods on the national list of essential medicines, to ensure availability within the public and private sectors

Accordingly, public health policies need to (1) ensure provision of a much wider range of contraceptive methods; (2) improve the efficiency of the supply chain distribution (with adequate monitoring and evaluation); and (3) strengthen demand generation activities. These actions will have a greater impact if they are developed within a context of strong political will, social accountability, and significant and sustained human and financial resources devoted to improve family planning services for the benefit of all women and their partners.

## Notes

**Peer Reviewed**

**First Published Online:** 2017 Feb 13

## References

[1] Lacina B Gleditsch NP Monitoring trends in global combat: a new dataset of battle deaths. Eur J Popul. 2005; 21(2-3):145–166. 10.1007/s10680-005-6851-6

[2] Muzima JD Mendy FR African Economic Outlook 2015: Regional Development and Spatial Inclusion. Paris: African Development Bank, Organisation for Economic Co-operation and Development, and United Nations Development Programme; 2015 http://www.africaneconomicoutlook.org/sites/default/files/content-pdf/AEO2015_EN.pdf Accessed January 31, 2017.

[3] Agadjanian V Prata N War and reproduction: Angola's fertility in comparative perspective. J South Afr Stud. 2001; 27(2):329–347. 10.1080/03057070120050000

[4] Instituto Nacional de Estatística (INE). Censo 2014: Resultados Definitivos do Recenseamento Geral Da População e da Habitação de Angola 2014. Luanda, Angola: INE; 2016 http://aiangola.com/wp-content/uploads/2016/03/Publica%C3%A7%C3%A3o-Resultados-Definitivos-Censo-Geral-2014_Vers%C3%A3o-22032016_DEFINITIVA-18H17.pdf Accessed January 31, 2017.

[5] Instituto Nacional de Estatística (INE). Integrated Survey on the Welfare of the Population, Vol. 1 Luanda, Angola: INE; 2011 http://washwatch.org/uploads/filer_public/44/9a/449aab71-2a32-4349-8158-8edc4699fc5e/wash_and_sanitation_strategy_angola_2011.pdf Accessed January 31, 2017.

[6] Cosep Consultoria, Consaúe, ICF Macro. Inquérito de Indicadores de Malária em Angola de 2011. Calverton, Maryland: Cosep Consultoria, Consaúde, and ICF Macro; 2011 http://dhsprogram.com/pubs/pdf/MIS10/MIS10.pdf Accessed January 31, 2017.

[7] Kuruvilla S Schweitzer J Bishai D; Success Factors for Womens and Childrens Health study groups. Success factors for reducing maternal and child mortality. Bull World Health Organ. 2014; 92(7):533–544. 10.2471/BLT.14.138131. 25110379 PMC4121875

[8] World Health Organization (WHO). Global Health Observatory Data [database online]. Geneva: WHO; 2015 http://who.int/gho/en/ Accessed September 14, 2015.

[9] United Nations Population Fund. Draft Country Programme Document for Angola. Geneva: UNFPA; 2014.

[10] Prata N Bell S Fraser A Carvalho A Neves I Andrade B Partner support for family planning and modern contraceptive use in Luanda, Angola. Paper presented at: Population Association of America Annual Meeting; April 30-May 2, 2015; San Diego, CA http://paa2015.princeton.edu/abstracts/152952 Accessed January 31, 2017. 10.29063/ajrh2017/v21i2.5 29624938

[11] RamaRao S Lacuesta M Costello M Pangolibay B Jones H. The link between quality of care and contraceptive use. Int Fam Plan Perspect. 2003; 29(2):76–83. 10.2307/3181061. 12783771

[12] Upadhyay UD Informed choice in family planning: helping people decide. Population Reports, Series J, No. 50. Baltimore, MD: The Johns Hopkins University Bloomberg School of Public Health; 2001 https://www.k4health.org/sites/default/files/j50.pdf Accessed January 31, 2017. 11552404

[13] Delivering for consumers: why is competition policy important for consumers? European Commission. http://ec.europa.eu/competition/consumers/why_en.html. Last updated April 16, 2012 Accessed July 24, 2016.

[14] United Nations Department of Economic and Social Affairs (UN DESA). Reproductive rights. New York: UN DESA; 2015.

[15] Agadjanian V Prata N Trends in Angola's fertility. New York: United Nations Department of Economic and Social Affairs; 2001 http://www.un.org/en/development/desa/population/events/pdf/expert/3/agadjani.pdf Accessed January 31, 2017.

[16] World Bank. Angola: reproductive health at a glance. Washington, DC: World Bank; 2011 http://documents.worldbank.org/curated/en/509271468193464621/Angola-Reproductive-health-at-a-glance Accessed January 31, 2017.

[17] Pathfinder International. Advancing contraceptive security in Angola. Luanda, Angola: Pathfinder International; 2015.

[18] Population Services International (PSI)/Angola. Barriers and drivers on family planning. Insights for developing a behavioral change communication program to increase demand for family planning services. Luanda: PSI/Angola; Forthcoming 2016.

[19] Ndomba B Faltam medicamentos nos hospitais püblicos. Rede Angola. Journalismo Independente. http://www.redeangola.info/especiais/faltam-medicamentos-nos-hospitais-publicos/. Published February 12, 2016 Accessed January 31, 2017.

[20] United Nations Fund for Population Activities, United States Agency for Development. List of donated contraceptives to Angolan Ministry of Health 2014-2015 [unpublished data].

[21] Gutierrez-Amo E Assessment of the family planning environment in Angola. Luanda, Angola Population Services International and United States Agency for International Development; 2012.

[22] United States Agency for International Development (USAID). Angola country development cooperation strategy 2014-2019. Washington, DC: USAID; 2014 https://www.usaid.gov/sites/default/files/documents/1870/Angola_CDCS_2014-2019.pdf Accessed January 31, 2017.

[23] ICF International. Demographic and Health Surveys Methodology–Questionnaires: Household, Woman's, and Man's. Calverton, Maryland: MEASURE DHS Phase III; 2011 http://countdown2030.org/documents/Country_workshops/DHS6_Questionnaires_3Jan2012.pdf Accessed January 31, 2017.

[24] Capo-Chichi V Chapman S Sampling strategies. PSI Research Toolkit. Washington, DC: Population Services International; 2007 https://www.k4health.org/sites/default/files/Sampling-Strategies-Toolkit.pdf Accessed January 31, 2017.

[25] Rehle T Saidel T Mills S Magnani R, eds. Evaluating Programs for HIV/AIDS Prevention and Care in Developing Countries: A Handbook for Program Managers and Decision Makers. Washington, DC: Family Health International; 2001 http://hivhealthclearinghouse.unesco.org/sites/default/files/resources/HIV%20AIDS%2082e.pdf Accessed January 31, 2017.

[26] Currency converter. OANDA Corporation. https://www.oanda.com/currency/converter/ Accessed October 31, 2014.

[27] Kopp Kallner H Thunell L Brynhildsen J Lindeberg M Gemzell Danielsson K. Use of contraception and attitudes towards contraceptive use in Swedish women: a nationwide survey. PLoS One. 2015; 10(5):e0125990. 10.1371/journal.pone.0125990. 25992901 PMC4439158

[28] Decker M Constantine NA. Factors associated with contraceptive use in Angola. Afr J Reprod Health. 2011; 15(4):68–77. 22571108

[29] KeyResearch. Estudo OMNIBUS: relatorios sobre componente de planejamento familiar. Luanda, Angola: KeyResearch; 2015 [unpublished].

[30] Ross J Hardee K Mumford E Eid S Contraceptive method choice in developing countries. Int Fam Plan Perspect. 2002; 28(1):32–40. 10.2307/3088273

[31] Instituto Nacional de Luta Contra a Sida (INLS), Joint United Nations Programme for HIV/AIDS (UNAIDS). Relatorio de UNGASS. Luanda, Angola: INLS and UNAIDS; 2007.

[32] PSI/Angola. Center for Health Market Innovations. http://healthmarketinnovations.org/program/psiangola Accessed July 21, 2016.

[33] United Nations Population Fund (UNFPA), Angolan Ministry of Health (MINSA). Contraceptivos na saúde reprodutiva: estudo nas províncias de Kwanza Norte e Kwanza Sul, 2010. Luanda, Angola: UNFPA and MINSA; 2010 http://angola.unfpa.org/sites/default/files/pub-pdf/contraceptivos_sr.pdf Accessed January 31, 2017.

[34] Addison S Miller DR Goredema W Analysis of the Angolan public health supply chain system. Arlington, VA: Management Sciences for Health, Systems for Improved Access to Pharmaceuticals and Services (SIAPS) Program; 2013 http://siapsprogram.org/tag/cecoma/?post_type=publication Accessed January 31, 2017.

[35] United Nations Development Programme (UNDP). Human Development Report 2015: Work for Human Development. New York: UNDP; 2015 http://hdr.undp.org/sites/default/files/2015_human_development_report.pdf Accessed January 31, 2017.

[36] Patrick M Angola cuts 2016 spending by 20%: country can cope with low oil prices, finance minister says. The Wall Street Journal. 3 14, 2016 http://www.wsj.com/articles/angola-cuts-2016-spending-by-20-1457980425 Accessed January 31, 2017.

[37] AFP. Angola's ailing national health system hit by oil price cuts. Times Live. Published May 16, 2016. http://www.timeslive.co.za/africa/2016/05/16/Angolas-ailing-national-health-system-hit-by-oil-price-cuts Accessed January 31, 2017.

[38] Angolan Ministry of Health (MINSA). Plano de necessidades de contraceptivos para 2016: Janeiro-Dezembro. Luanda, Angola: MINSA; 2015.

[39] Bell S Weidert K Vohra D Harris L Prata N Community survey report: Cacuaco and Viana Municipalities, Luanda, Angola. Berkeley, CA: Bixby Center for Population, Health and Sustainability, University of California, Berkeley; 2012 http://bixby.berkeley.edu/wp-content/uploads/2015/04/Angola-Community-Level-Assessment-Report-FINAL.pdf Accessed January 31, 2017.

